# Effect of primary health care on hospitalizations: health services analysis based on Estonian data

**DOI:** 10.1017/S1463423625000222

**Published:** 2025-03-20

**Authors:** Kaija Kasekamp, Andres Võrk, Ruth Kalda

**Affiliations:** 1 Institute of Family Medicine and Public Health, PhD student, MA in Financial Management and Accounting, University of Tartu, Tartu, Estonia; 2 Faculty of Social Sciences, Johan Skytte Institute of Political Studies, Analyst, MA in Economics, University of Tartu, Tartu, Estonia; 3 Faculty of Medicine, Institute of Family Medicine and Public Health, Professor of Family Medicine, Doctor of Medical Science, University of Tartu, Tartu, Estonia

**Keywords:** primary health care, comorbidity, hospitalization, delivery of health care, Estonia

## Abstract

**Aim:**

This study aims to assess the effect of primary health care (PHC) service provision continuity on inpatient admissions for people with chronic diseases in Estonia.

**Background:**

Non-communicable diseases (NCDs) were collectively responsible for more than 7 out of 10 deaths worldwide in 2019. As the burden of NCDs increases, PHC has an increased role of coordinating care management. High-performing PHC can reduce unnecessary hospitalizations. Estonia has a strong PHC system focusing on multidisciplinary care. Yet it has not been evaluated for its effect on hospitalizations. Therefore, it is imperative to evaluate PHC continuity to improve care for NCD patients.

**Methods:**

This study used routinely collected electronic medical billing data of the Estonian population aged 15 years or older from 2005 to 2020 identifying patients with seven ambulatory care sensitive chronic (ACSC) conditions. We developed an indicator to describe the continuity of PHC. Charlson Comorbidity Index (CCI) was used to assess the impact of comorbidities and we controlled the patient’s age, gender, county of residency and socio-economic status. We estimated multilevel logistic regression models with family doctor patient list random effects to assess how the odds of hospitalization depend on continuity of care, allowing for confounders.

**Findings:**

We identified that 45% of the adult Estonian population had at least one of the target diagnoses. Among the target population, 96% had contact with their PHC providers. We found that there is a non-linear relationship between PHC continuity and patient outcomes. Any contact with PHC provider during the past 5 years decreases odds for hospitalization, but hospitalization risk is higher for people who are elderly and have higher CCI score. We found that after accounting for patient characteristics, differences among patient lists minimally impact outcomes. Further research should explore policies to better support family doctors in reducing hospitalizations for chronic patients.

## Introduction

According to the World Health Organization, Non-communicable diseases (NCDs)^1^ are collectively responsible for almost 71% of all deaths worldwide in 2019, or 41 of the total 55 million deaths. (World Health Organization, 2022). Ensuring adequate treatment of patients with NCDs, also known as chronic diseases, plays an important role in the epidemiological and population health perspective. The increase in the number of patients with multiple co-morbidities leads to a greater need for health care (Ansari *et al.*, 2006; Lee *et al.*, 2015).

Primary health care (PHC) plays a central role in coordinating the treatment of patients with NCDs and multiple chronic conditions (Van den Akker *et al.*, 1998; Wilkinson & Marmot, 2003). As the global burden of NCDs increases, PHC is taking the burden of coordinating life-long management of chronic conditions (Hanson *et al.*, 2022). High-performing PHC has been regularly found to reduce unnecessary hospitalizations and costly emergency room visits, offering cheaper and better management of high-prevalence chronic conditions with unit costs below those that apply in higher-level health facilities (OECD, 2020). In a health system with appropriate access to and effective provision of PHC, hospital admissions for ambulatory care sensitive chronic (ACSC) conditions should largely be avoidable (Caminal *et al.*, 2004). Studies have shown that the probability of an inpatient treatment episode is lower if the patient has visited a family doctor (Atun *et al.*, 2016). Therefore, improving PHC systems has significant potential to ease the burden of healthcare systems imposed by increasing numbers of chronically ill patients.

We aim to contribute to available evidence by analysing PHC continuity for people with core chronic conditions and its impact on adverse effects. Estonia is a good example of a strong PHC system having successfully implemented PHC reforms, including new organizational structures, user choice of family doctors, new payment methods, specialist training for family medicine, service contracts for family doctors, broadened scope of services, and evidence-based guidelines. Furthermore, all these changes have been institutionalized (Kasekamp *et al.*, 2022). Patients need a referral from family doctor to access secondary outpatient care or inpatient care for the majority of health conditions. Patients can freely select their preferred family doctor at any point in time.

Estonia has implemented since 2006 a quality improvement program of family doctors (quality bonus system, QBS) to incentivize improving care continuity for patients with core ACSC conditions. The family doctors in Estonia are owners of a patient list and the QBS aims to assess whether the patients enrolled to a specific doctor patient list receive care in alignment with clinical guidelines which define the recommended contacts for PHC. So far, no scientific research has been conducted in Estonia that would comprehensively assess the impact PHC continuity to manage chronic patients. There is limited scientific evidence on the QBS program monitoring service delivery alignment with clinical guidelines and impact on provider resource needs (Merilind, 2016; Merilind *et al.*, 2017). This study aims to fill the gap and is timely in providing input for policy changes in PHC organization helping policy-makers to make better informed decisions while improving service delivery models and financing to adapt to the challenges of increasing burden of NCDs.

## Methods

### Data sources and collection

We used routinely collected electronic medical billing data from Estonian Health Insurance Fund (EHIF) and Estonian Causes of Death Registry (ECDR) information for the years of 2005–2020. The electronic medical bills include comprehensive data on patient characteristics and services utilization. Estonia has one national health insurance fund drawing together medical bills for the entire insured population (94% of the total population) (European Observatory on Health Systems and Policies, 2021). PHC and inpatient care in Estonia is mostly provided by EHIF funded providers (Kasekamp *et al.*, 2023). Less than 1% of inpatient care services were funded outside of the mandatory health insurance system in 2020 (National Institute of Health Development, 2022). This limits the risk of selection bias of excluding patients paying for care privately or leaving out uninsured population groups. For the population socioeconomic status, the EHIF insurance registry was used, which describes the person’s entitlement for insurance depending on employment and receipt of social security payments. The patients’ date of death was retrieved from the ECDR.

The quality of the data is expected to be high because EHIF uses an electronic invoicing system which checks all submitted bills to have appropriate patient data, diagnoses, and other relevant information related to the contact with the health system. In addition, EHIF uses a retrospective analytical reporting system to identify systematic outliers that are not possible to detect during billing. In addition, the data submitted to EHIF by providers can also be monitored by the patients through the national patient portal (WHO, 2023). Therefore, we expect a minimum level of under-or overreporting.

### Target group

The target group includes all individuals 15 years of age and older. We selected patients with ACSC conditions which should be effectively managed in PHC (Solberg *et al.*, 1990; Weissman *et al.*, 1992; Pinto *et al.*, 2020). The selected diseases cause a proportionately high disease burden in Estonia (Lai *et al.*, 2009). The selection of key ACSC conditions was based on availability of clear treatment protocols that have been described in previous studies (Caminal *et al.*, 2004; Purdy *et al.*, 2009; Atun *et al.*,^2016^). This study adds depression to the ACSC condition list because it causes significant permanent disability due to illness (Atun, 2015).

We used population-level data selecting every person with at least one of the seven ACSC diagnoses described on any medical bill from 2005 to 2020 to the sample. We considered primary diagnosis and concomitant diagnoses. The medical bills of the EHIF are coded with diagnostic information using the 10th edition of the International Classification of Diseases (ICD-10). The sample includes medical bills from all patients of the following diseases listed in Table 1.

**Table 1.** ACSC

PHC-sensitive conditions	ICD-10 code
Ischaemic heart disease (IHD)	I20 & I25
Heart failure	I50
Adult asthma (asthma)	J45
Non-insulin dependent diabetes mellitus (type 2 diabetes)	E11
Depression	F32
Chronic obstructive pulmonary sisease (COPD)	J44.9
Hypertension	Il0 & I11-I15

In total, 723,758 persons data were extracted from the EHIF database including characteristics of individuals such as gender, county of residence, and age, divided into 5 groups (15 to 45, 45 to 55, 55 to 65, 65 to 75, and over 75). The age groups are purposefully selected to consider the increase in disease burden among the older population. Socioeconomic status is grouped into 5 categories of employed, unemployed, old-age pensioner, disability pensioner, other, and people with no information which means that they are not covered with health insurance. The socioeconomic status is retrieved at the end of each year unless the person had died during the year. In this case, the previous year’s status is used. The patient data also include information on enrollment to a PHC provider patient list at the end of the year, which is used as a random effect in multilevel model.

## Data and analysis methodology

The analysis was conducted with pseudonymized data to prevent possible identification of the target population. Following data cleaning, in total, 723,492 persons remained in the sample. Hospitalization data were monitored for each person in the sample for the relevant ACSC conditions. We also looked at the patient list identifier where the patient is registered to in a given year as a random effect in a multilevel model to capture the effect of unobserved provider-level characteristics on hopsitalizalisation. To understand the association of the PHC continuity with hospitalizations, we created a dataset of patient-year observations. We considered only medical bills that followed the patient’s first diagnosis with an ACSC condition. Each observation in the dataset included information listed in Table 2.

**Table 2.** Description of variables

Domain	Variable	Values
Characteristics of the exposure	Continuity of PHC visits (fraction of previous five years with PHC visit)	0
		0.2
		0.4
		0.6
		0.8
		1
Outcome characteristics	Hospitalization for the ACSC	No
		Yes
Confounders	Comorbidity, CCI	0
		1 to 3
		Over 3
	Age	15–44
		45–54
		55–64
		65–74
		75+
	Sex	Male
		Female
	Settlement	County of residence (15)
	Socioeconomic status	Employed
		Unemployed
		Old-age pensioner
		Disability pensioner
		Other
		No information (uninsured)

We developed a variable to describe the contact continuity of care based on the service utilization. Care is continuous over time if it involves the relationship between the health workers and patients built on trust, loyalty, and constancy of an individual patient (Khatri *et al.*, 2023). Care continuity and coordination has proven effects for health outcomes (D’haenens *et al.*, 2020). Though, with this variable we do not assume that the continuous contacts have to be done by the same family doctor as we focus on the patient-level characteristics describing how often the patient has contact for their underlying condition. For the variable, we use a fraction of years with PHC contacts^2^ during past 5 years non depending on the cause of the contact^3^ as it is more informative. We assume that higher fractions represent better continuity of care. The PHC continuity variable prevented us from using data prior to 2010 in the final analysis.

We used the Charlson comorbidity index (CCI) (Charlson *et al.*, 1987) as an indicator capturing the effect of health status of the patient. When adjusting for comorbidities, we decided to use a summary measure instead of the effect of single comorbidities, because we aimed not to look at the individual impact of each condition but rather the general health condition of the patient. We used the revised coding algorithm described by Quan, *et al.* (2005) and identified CCI based on diagnoses in any medical bill for primary, outpatient, or inpatient care. CCI score is calculated for every year based on the previous 5-years medical bills data.

Since the patient’s age changes over time, we defined it at the beginning of the year. We considered the clustered nature of the dataset by controlling for the random effect of patient list. In addition, we added the patient’s county of residence to the models to capture regional differences in the supply of health care services. The county of residence is considered according to information described on medical bills. If the county of residence was missing, we used the most commonly defined county from the medical bills. The belonging to a specific patient list was defined at the end of the year. The outcome variable is hospitalization. Only hospitalizations for relevant ACSC conditions were considered.

Using patient-level data, we first visualized the association of inpatient admissions with PHC continuity of care for the years of 2010, 2015, and 2020. To test the sensitivity and significance of the obtained results, we estimated a multilevel logistic regression model for annual datasets of 2010, 2015, and 2020 looking to assess the odds of a hospitalization for target population and checking for the listed confounders. We also tested the results for the complete study period including data from 2010 to 2020 allowing us to see the trend throughout this period. The odds ratios (ORs), its confidence intervals (CId) with the statistical significance indicator as p-value is presented. The data analysis was conducted in statistics software Stata.

## Results

The number of target population with at least one of the seven ACSC conditions (table 1) accessing health services per year increased from 260,000 to 500,000 corresponding to 23% and 45% of Estonian population aged 15 years and older from 2005 to 2020. The increase may be explained by improved diagnostics, coding and reporting practices, but as well population aging and increase in the prevalence of these conditions. Around 61% of the target population using health services were female.

On average, 95% of the patients contacted their PHC provider, 72% outpatient specialist care services, and 21% had inpatient admissions during the period 2005–2020. The share of patients contacting PHC among the target population has remained 96%, and there has been a 1% reduction in the number of patients with inpatient admissions from 2005 to 2020 (Table 3). 64% of the target population had hypertension, 13% type 2 diabetes, 14% ischaemic heart disease, 12% heart failure, 7% asthma and depression, and 1 % chronic obstructive pulmonary disease (COPD). The disease profile has not changed from 2005 to 2020 for COPD patients and asthma. For type 2 diabetes, there has been an increase and for other conditions, there has been a decline.

**Table 3.** Patient characteristics for the research variables in 2005, 2010, 2015, and 2020

		2005	2010	2015	2020
Hospitalization	No	88%	89%	90%	91%
	Yes	12%	11%	10%	9%
PHC contact on previous year	No	–	8%	7%	6%
	Yes	–	92%	93%	94%
PHC continuity	0	–	8%	7%	6%
	0.2	–	1%	2%	2%
	0.4	–	3%	4%	3%
	0.6	–	6%	7%	6%
	0.8	–	11%	11%	10%
	1	–	71%	69%	73%
Age group	15-44	13%	20%	21%	21%
	45-54	14%	16%	15%	14%
	55-64	21%	22%	21%	20%
	65-74	27%	21%	20%	22%
	75+	24%	21%	23%	23%
Sex	Male	35%	39%	41%	42%
	Female	65%	61%	59%	58%
CCI	0	–	53%	56%	58%
	1 to 2	–	31%	29%	27%
	3+	–	16%	15%	15%
Socioeconomic status	Employed	23%	25%	27%	27%
	Unemployed	1%	3%	2%	2%
	Old-age pensioner	62%	52%	50%	48%
	Other	4%	4%	4%	4%
	Disability pensioner	8%	10%	12%	12%
	No info	2%	4%	6%	5%

**Figure 1.**
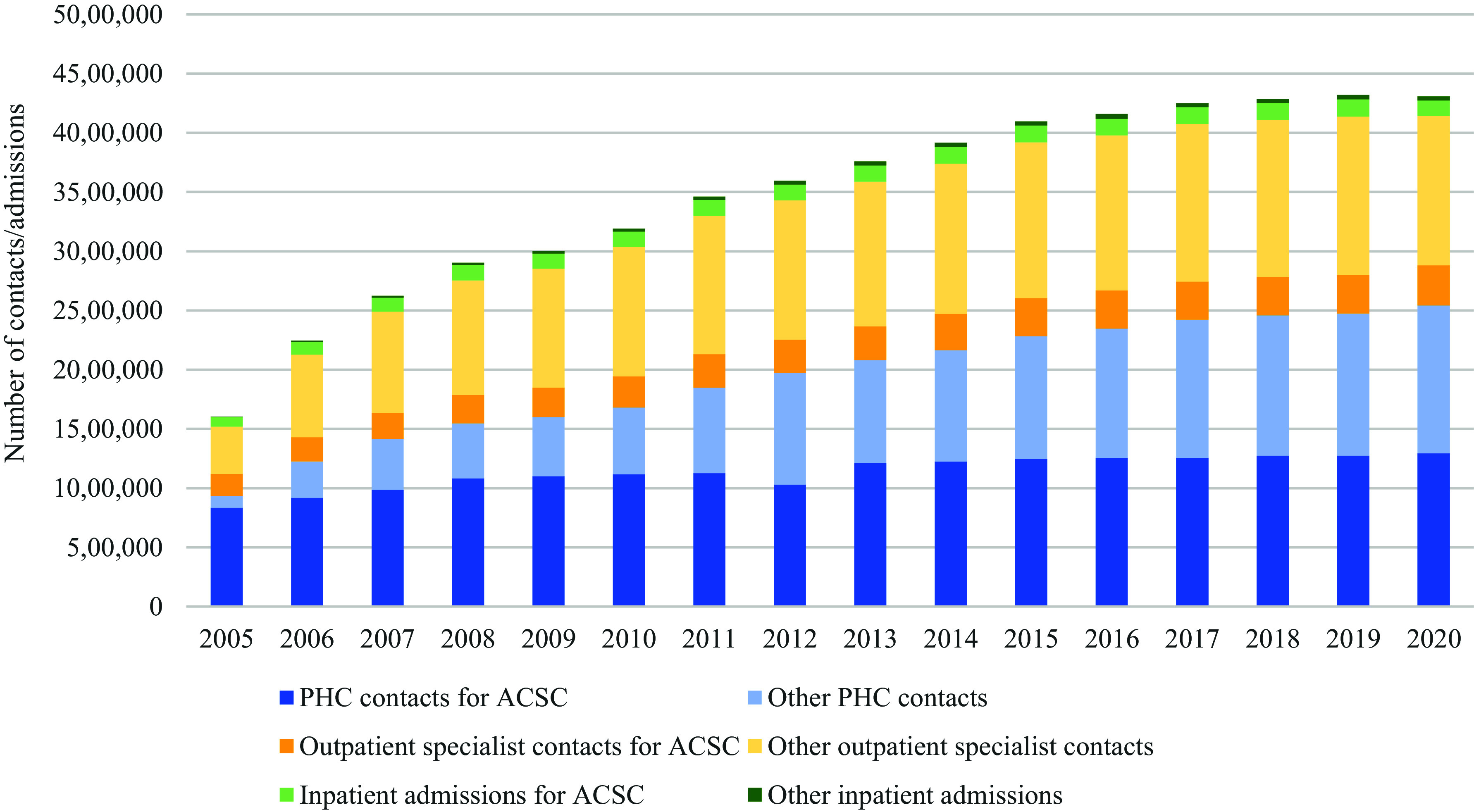
Service utilization among target population from 2005 to 2020.

The number of contacts conducted because of ACSC conditions increased from 1.1 million in 2005 to 1.8 million in 2020. If to consider all contacts (including contacts for ACSC condition but also for other conditions), the increase has been from 1.6 million to 4.3 million from 2005 to 2020. Figure 1 depicts the changes in the utilization pattern from 2005 to 2020. Most of the contacts are conducted at the PHC level, especially for ACSC conditions, although the share of PHC contacts for the ACSC conditions out of all contacts has decreased from 75% in 2005 to 73% in 2020. Compared to 2005, the share of ACSC condition-related contacts in all patient contacts has also decreased from 69% to 41% in 2020 indicating that people with ACSC condition contact healthcare providers more for other reasons than their chronic condition, but this may be an impact of changes in reporting practice as the reporting requirements for PHC contacts has been revised several times through the course of the study period allowing to record nurse consultations and remote consultations.

We compiled the dataset of patient year panel data including over 7.4 million observations to illustrate changes in patient characteristics and utilization from 2005 to 2020 (Table 3). The age structure of the target population has changed with an increased number of patients in the youngest age group of 15 to 44. The share of men in the target population has increased. Most of the target population was identified to have the lowest index for CCI. The analysis indicates that the severity of patients’ health conditions has declined with fewer people having higher rates of CCI. The socioeconomic status of the target population has also significantly changed from 2005 to 2020. The share of pensioners has significantly declined, but on the other hand, the share of the population with disability pensions has increased – this is a result of a pension policy reform which moved some people priorly grouped as pensioners to disability pension group. There is an increase in the share of employed. The share of persons with the socioeconomic status “other” has remained the same. The share of unemployed people has slightly increased as well as the share of the population whose status is unknown (Table 3).

The number of overall inpatient admissions has declined significantly by 2020 in comparison to 2005. In 2010, 71% of patients had contacted a PHC provider every year in the past five years, in 2020 the share is 73%. The share of patients not having visited their PHC provider for their ACSC condition has declined from 8% in 2010 to 6% in 2020.

**Figure 2.**
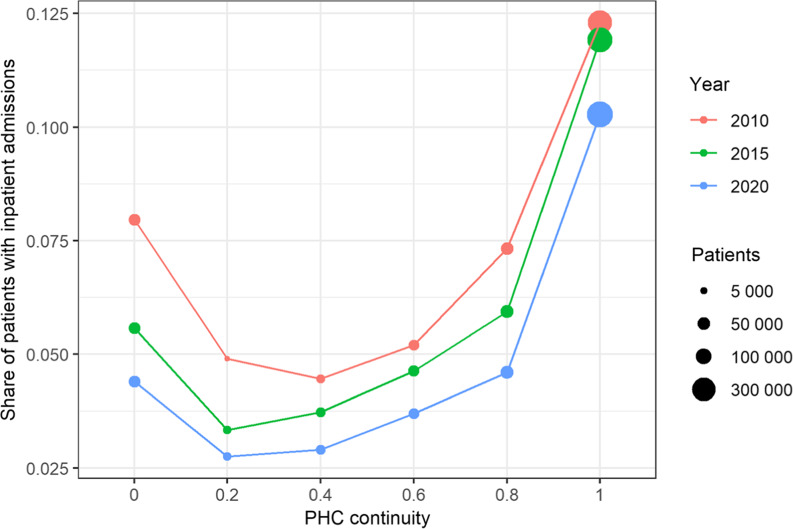
PHC care continuity association with inpatient admissions in 2010, 2015, and 2020 for total target population.

We visualized the association of PHC continuity and inpatient admissions for years 2010, 2015, and 2020 (Figure 2). For all three years (2010, 2015, and 2020), the relationship between PHC continuity and the share of inpatient admissions follows a similar U-shaped curve and is nonlinear. Therefore, we also include the variable into the regression model as a set of indicator variables to allow for the nonlinear effects. Patients who annually visit their PHC provider have more hospitalizations. Patients with no PHC provider contacts during the past five years have increased numbers of inpatient admissions. Patients who have at least 1 to 4 visits to PHC providers during past five years have fewer inpatient admissions. Over time, the share of inpatient admissions has decreased, particularly at the lower end of PHC continuity and the larger bubbles appear at the higher end of PHC continuity, indicating a larger number of patients with higher PHC continuity (Figure 2).

We applied a multilevel logistic regression model to assess the statistical significance of the results and to examine the odds of inpatient admissions for patients that accessed health services in 2010, 2015, and 2020 for any condition, controlling for the different personal confounders and allowing for random effects of PHC patient list (Table 4). Being male, elderly, and having a higher previous CCI score has a significant impact on increasing the odds of hospitalization. Also, the odds of hospitalization are increased for old-age pensioners, disability pensioners and unemployed people compared to employed persons. The patients not visiting a PHC provider at least once during the past 5 years have increased odds for hospitalization compared to patients who visit PHC provider at least once during past 5 years. The odds for hospitalization are lower for patients who visit a PHC provider 1 to 4 times during past years compared to patients who have annual visits. This may be explained by sicker patients requiring more services. It is evident that the hospitalizations are mostly influenced by person‘s age and CCI score (Table 4). We looked at the variance between patient lists. The low values of intraclass correlation coefficient and between patient list variance in the full model, and the reduction of variance compared to the empty model, suggest a relatively low clustering effect (Table 4). Most of the variation in the outcome is due to individual-level factors rather than group-level differences. Once patient characteristics are considered, patient list-level differences contribute minimally to the outcome. Still, the between-patient list level variance remains statistically significant in our multilevel model.

**Table 4.** Multilevel logistic regression results for inpatient admission likelihood for 2010, 2015 and 2020 data

Variable	2010 OR (95% CI)	2015 OR (95% CI)	2020 OR (95% CI)
**PHC coverage (ref: 1)**			
0	1.845*** [1.764, 1.930]	1.551*** [1.473, 1.634]	1.636*** [1.544, 1.733]
0.2	0.783** [0.663, 0.924]	0.768*** [0.692, 0.853]	0.835** [0.743, 0.938]
0.4	0.732*** [0.673, 0.796]	0.730*** [0.676, 0.788]	0.733*** [0.671, 0.802]
0.6	0.757*** [0.715, 0.802]	0.789*** [0.747, 0.833]	0.810*** [0.760, 0.863]
0.8	0.852*** [0.821, 0.884]	0.805*** [0.775, 0.836]	0.815*** [0.780, 0.851]
**Gender (ref: male)**			
Female	0.730*** [0.715, 0.745]	0.749*** [0.734, 0.764]	0.703*** [0.689, 0.718]
**Age group (ref: 15–44)**			
Age 45–54 years	1.943*** [1.829, 2.063]	1.806*** [1.701, 1.919]	1.914*** [1.795, 2.041]
Age 55–64 years	2.487*** [2.343, 2.638]	2.313*** [2.184, 2.449]	2.503*** [2.358, 2.657]
Age 65–74 years	3.547*** [3.308, 3.803]	3.218*** [2.999, 3.453]	3.492*** [3.222, 3.785]
Age 75+ years	5.211*** [4.861, 5.585]	5.165*** [4.816, 5.539]	5.815*** [5.366, 6.300]
**Comorbidity according to CCI score (ref: 0)**			
CCI score 1-2	2.122*** [2.066, 2.180]	2.005*** [1.953, 2.057]	1.919*** [1.869, 1.970]
CCI score 3+	4.108*** [3.993, 4.227]	4.001*** [3.894, 4.111]	3.815*** [3.713, 3.921]
**Socioeconomic status (ref: employed**			
Unemployed	1.080 [0.980, 1.190]	1.108 [0.960, 1.279]	1.203** [1.067, 1.356]
Old-age pensioner	1.887*** [1.789, 1.991]	1.999*** [1.891, 2.113]	2.040*** [1.908, 2.180]
Other	0.891 [0.792, 1.003]	0.992 [0.885, 1.112]	1.149* [1.029, 1.283]
Disabled	2.917*** [2.787, 3.053]	2.756*** [2.635, 2.882]	3.017*** [2.879, 3.161]
No info	0.550*** [0.479, 0.632]	0.470*** [0.409, 0.539]	0.436*** [0.372, 0.511]
**Random effect**			
Var [patient list]	0.026*** [0.021, 0.031]	0.021*** [0.017, 0.025]	0.020*** [0.016, 0.024]
ICC	0.008	0.006	0.006
LR test (ML vs Logit)	466.0 (p = 0.000)	332.4 (p = 0.000)	316.4 (p = 0.000)
Observations	452 932	514 056	545 420
Groups	808	806	789
Var [patient list] in an empty model	0.133*** [0.116, 0.152]	0.137***[0.121, 0.156]	0.148***[0.130, 0.168]


OR – odds ratio; 95% CI – confidence interval; ICC – intraclass correlation coefficient.

statistical significance as p-value * p < 0.05, ** p < 0.01, *** p < 0.001.

We also estimated the model for the pooled dataset from 2010 to 2020 (Table 5) including 5.6 million observations. Through the course of 10 years, similar outcomes were seen as for yearly observations. The odds of inpatient admission were smaller for females. Patients older than 75 years were more than five times more likely to be hospitalized compared to patients in the age group 15 to 44. Patients with a CCI score of three or more were almost four times more likely to be hospitalized compared to patients with a CCI score of zero. Those who are unemployed, pensioners, disability pensioners have an increased odds of hospitalizations compared to the employed population. Uninsured people were less likely to be hospitalized compared to the employed population, which may be an effect of working-age people having no coverage. Statistically significant increased odds of inpatient admissions compared to Harju County were observed in Hiiu, Jõgeva, Lääne, Pärnu, Rapla, Saare, and Viljandi counties. Also, the pooled data analysis confirms that if there is no PHC contact during the past 5 years it significantly increases odds for hospitalization. More frequent monitoring of a patient by a PHC provider can be associated with a higher number of hospitalizations. The overall time trends indicate a decline in odds for hospitalizations in 2020 compared to 2010. A more significant decline has been noticed for 2020, which may be caused by the decline in hospitalization numbers due to the COVID-19 pandemic impact. The variation explained by random effects at patient list level is statistically significant, but its contribution to the variability of inpatient admissions is very low compared to patients’ characteristics, which is confirmed by the reduction of variance in the full model compared to the empty model.

**Table 5.** Multilevel logistic regression results for inpatient admission likelihood

Variable	OR	CI	p-value
**PHC coverage (ref: 1)**			
0	1.577	[1.553, 1.602]	< 0.001 ***
0.2	0.778	[0.752, 0.805]	< 0.001 ***
0.4	0.749	[0.731, 0.767]	< 0.001 ***
0.6	0.770	[0.757, 0.784]	< 0.001 ***
0.8	0.814	[0.805, 0.824]	< 0.001 ***
**Gender (ref: male)**			
Female	0.738	[0.733, 0.742]	< 0.001 ***
**Age group (ref: 15–44)**			
Age 45–54 years	1.855	[1.822, 1.890]	< 0.001 ***
Age 55–64 years	2.343	[2.302, 2.384]	< 0.001 ***
Age 65–74 years	3.265	[3.195, 3.337]	< 0.001 ***
Age 75+ years	5.181	[5.070, 5.294]	< 0.001 ***
**Comorbidity according to CCI score (ref: 0)**			
CCI score 1-2	2.012	[1.996, 2.028]	< 0.001 ***
CCI score 3+	4.032	[3.999, 4.066]	< 0.001 ***
**Socioeconomic status (ref: employed)**			
Unemployed	1.179	[1.134, 1.225]	< 0.001 ***
Old-age pensioner	2.037	[2.002, 2.072]	< 0.001 ***
Other	1.047	[1.012, 1.083]	0.007 **
Disabled	2.898	[2.858, 2.938]	< 0.001 ***
No info	0.489	[0.468, 0.510]	< 0.001 ***
**Geographical region, county (ref: Harju)**			
Hiiu	1.259	[1.166, 1.359]	< 0.001 ***
Ida-Viru	0.987	[0.961, 1.014]	0.378
Järva	1.016	[0.974, 1.059]	0.460
Jõgeva	1.071	[1.031, 1.113]	< 0.001 ***
Lääne	1.234	[1.176, 1.294]	< 0.001 ***
Lääne-Viru	1.021	[0.988, 1.056]	0.200
Pärnu	1.099	[1.063, 1.136]	< 0.001 ***
Põlva	0.982	[0.940, 1.026]	0.401
Rapla	1.179	[1.133, 1.226]	< 0.001 ***
Saare	1.235	[1.172, 1.301]	< 0.001 ***
Tartu	1.025	[0.999, 1.052]	0.061
Valga	1.014	[0.971, 1.058]	0.547
Viljandi	1.107	[1.065, 1.151]	< 0.001 ***
Võru	1.022	[0.978, 1.067]	0.330
Unkown	0.278	[0.250, 0.311]	< 0.001 ***
**Billing year (ref: 2010)**			
2011	0.985	[0.971, 0.998]	0.031 *
2012	0.954	[0.941, 0.968]	< 0.001 ***
2013	0.930	[0.917, 0.943]	< 0.001 ***
2014	0.928	[0.915, 0.941]	< 0.001 ***
2015	0.909	[0.896, 0.922]	< 0.001 ***
2016	0.869	[0.857, 0.881]	< 0.001 ***
2017	0.862	[0.850, 0.874]	< 0.001 ***
2018	0.861	[0.849, 0.873]	< 0.001 ***
2019	0.863	[0.851, 0.875]	< 0.001 ***
2020	0.780	[0.769, 0.791]	< 0.001 ***
**Random effect**			
Var [patient list]	0.021	[0.019, 0.024]	
ICC	0.006		
Observations	5,590,146		
Groups	829		
LR Test (ML vs. Logit)-	7590.75		< 0.001 ***
Var [patient list] in an empty model	0.190	[0.172, 0.210]	

OR – odds ratio; 95% CI – confidence interval; ICC – intraclass correlation coefficient.

Statistical significance as p-value * p < 0.05, ** p < 0.01, *** p < 0.001.

## Discussion

Our analysis summarized the patterns of PHC and outpatient care utilization and inpatient admissions for ACSC patients over the course of 15 years. By 2020, over 45% of the Estonian population were registered in the healthcare system as having at least one ACSC condition. The outpatient contacts of the target population have significantly increased, requiring more resources from healthcare providers. Even when the COVID-19 pandemic starting in 2020 resulted in a drop in patient contacts from 2019 to 2020 in our target group, contact made because of the underlying ACSC condition still increased from 2019 to 2020. In order to sustain high-quality standards and align with the clinical guidelines, there is an increasing need for laboratory diagnostics and counselling population with ACSC conditions. This increased burden challenges the ability to sustain needed continuity of chronic patients’ management. The attempts to monitor care continuity mostly focus on processes, but there is no evidence on outcomes for patients with ACSC condition. That is why we evaluated whether care continuity is associated with hospitalizations for ACSC condition.

We found that the odds for hospitalization declined during the study period, indicating improved management of the patients with ACSC conditions. Previous analysis has shown that patients were less likely to have an inpatient admission if they had any PHC visit in the given year, suggesting a protective effect of PHC consultations (Atun *et al.*, 2016) Our analysis showed that any contact with the PHC provider during past five years will reduce odds for hospitalization, but there is nonlinear relationship between PHC continuity and patient outcomes, demonstrating that both ends of the continuity spectrum are associated with higher inpatient admissions. If the aim is to reduce of hospitalization, policies should be implemented to better support PHC providers to do outreach for patients who have not visited the PHC providers for a longer period. However, patients who visited their PHC provider annually were more likely to have an inpatient admission, reflecting a situation where sicker people need both PHC and hospital services more. This is an important finding to support the outcomes of previous studies highlighting the need to increase medical staff and funding to effectively manage chronic conditions at the PHC level in Estonia (Merilind, 2016) and need for establishing multidisciplinary PHC teams and increasing the role of family nurses (Habicht et al., 2022).

In 2015, an assessment on care integration in Estonia indicated that 18% of hospitalizations for asthma, COPD, type 2 diabetes, heart failure, and hypertension could have been prevented^4^ with more effective outpatient treatment (The World Bank, 2015). PHC is easily accessible and highly recognized among the population (Emor/ Estonian Health Insurance Fund, 2020), but health care has still been assessed to be too specialist care centered and PHC quality is uneven. (The World Bank, 2015) There are ongoing efforts to strengthen PHC to meet the needs of an aging population with increasing NCD burden (Kasekamp *et al.*, 2023), but no policies besides the QBS system have been put in place to improve care continuity of patients with core ACSC condition. We found that when controlling for patient characteristics, patient list-level differences contribute minimally to the outcome in Estonia. Future policy directions could focus utilizing the extensive database of EHIF and identifying patients with high probability of having an adverse health event. This would need to be accompanied with ensuring adequate resources for PHC providers to manage chronic patients proactively. There are several pilots conducted in Estonia to apply risk-based care management at PHC level, but none of the pilots have been scaled nationwide (SA Viljandi Haigla, 2020; The World Bank, 2017).

The comprehensive data used in this analysis have enabled robust analysis to provide a deeper understanding of health care utilization trends in Estonia and the effect of PHC continuity on hospitalizations of the target population. We acknowledge our study limitations that there are additional provider-level characteristics as well as specific policies, which may also impact hospitalizations for patients with core ACSC conditions like proximity of a hospital, access to PHC in rural areas, size of the PHC team, and number of patients in a list. As adding the provider-level characteristics would have limited the understanding on the impact of patient level, we decided to focus on patient level characteristics considering the impact of belonging to a specific patient list. Our analysis confirmed very little variance between the different patient list justifying the focus on patient level characteristics. Nonetheless, this information may not be useful for policy-makers who may seek for evidence on the impact of different health policies for making better informed decisions. Therefore, we still suggest a more detailed analysis to understand what policy changes and provider-level characteristic may have additionally contributed to reduction of hospitalization among the chronic patients.

## Conclusion

To conclude, the study provides evidence on increasing burden of PHC providers to manage patients with core ACSC condition. Our study findings indicate that there is a nonlinear relationship between PHC continuity and patient outcomes but any contact with PHC provider during the past 5 years significantly reduces odds for hospitalization for ACSC conditions. Those patients with poorer health require both more frequent visits to the family doctor and occasional hospitalization. If the aim is to reduce the burden of hospitalization, policies should be implemented to better support these patients by the PHC system. This is a relevant outcome for all countries seeking options to reduce the burden for the health system under the increasing demand due to the rise of NCDs.

## Footnotes

1 According to World Health Organization definition include heart disease, stroke, cancer, type 2 diabetes and chronic lung disease.

2 In Estonia, PHC contacts do include also services provided by family nurses and services provided remotely e.g. phone calls or e-mail contacts.

3 If for example 3 visits are done during the past five years, the indicator has value 0.6. It does not consider if the visits have been consecutive.

4 According to the OECD protocol, preventable hospitalisation is considered to be what could have been prevented by adequate outpatient treatment.
